# Supplementary material for: The Potential of Purple Waxy Corn Cob (Zea mays L.) Extract Loaded-Sericin Hydrogel for Anti-Hyperpigmentation, UV Protection and Anti-Aging Properties as Topical Product Applications
Source: Pharmaceuticals (Basel). 2022 Dec 27;16(1):35. doi: 10.3390/ph16010035 (PMC9864391; doi:10.3390/ph16010035)
Supplement: Supplementary file 1 [file pharmaceuticals-16-00035-s001.zip › pharmaceuticals-2099147-supplementary.pdf]

Supplementary materials

Table S1: Structure of anthocyanin derivatives and calculated molecular ion and fragment ion for MS detection.

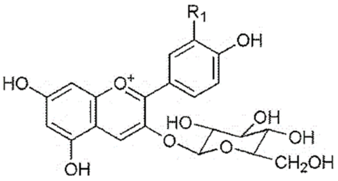

Anthocyanin	R1	*[M+H]*+ (*m/z*)	Fragments ions (*m/z*)
Cyanidin-3-*O*-glucoside (C3G)	OH	449	287
Peonidin-3-*O*-glucoside (Pn3G)	OCH_3	463	301
Pelagonidine-3-*O*-glucoside (Pg3G)	H	433	271

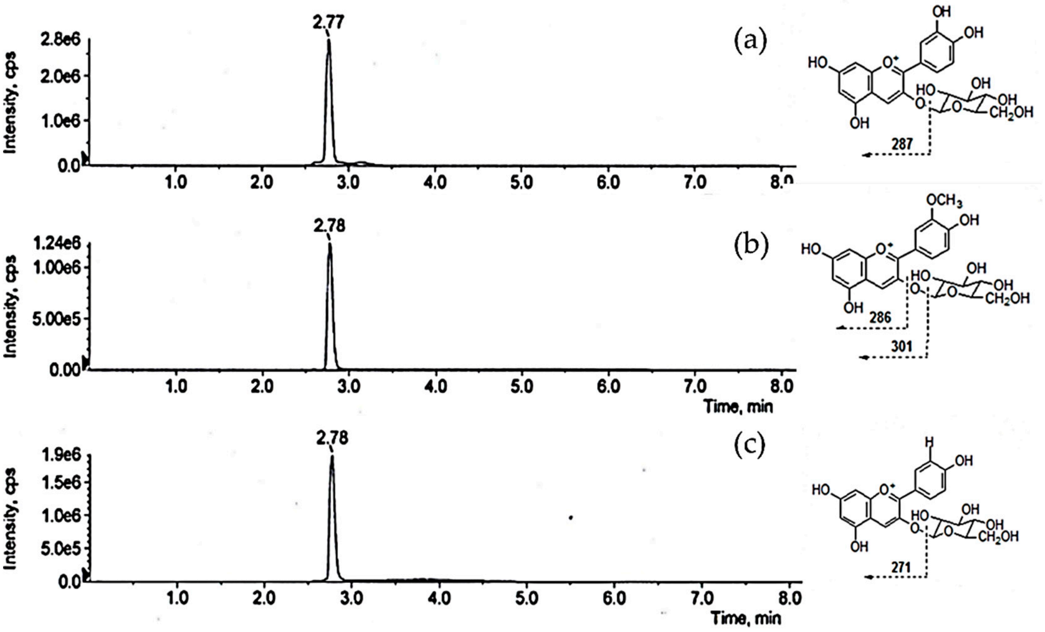

Figure S1: LC-MS/MS chromatogram of anthocyanin from purple corn cob 1 µg/mL extracted by computer software to C3G (449/287) (a), Pn3G (463/301) (b), Pg3G (433/271) (c), retention time 2.77-2.78 min.

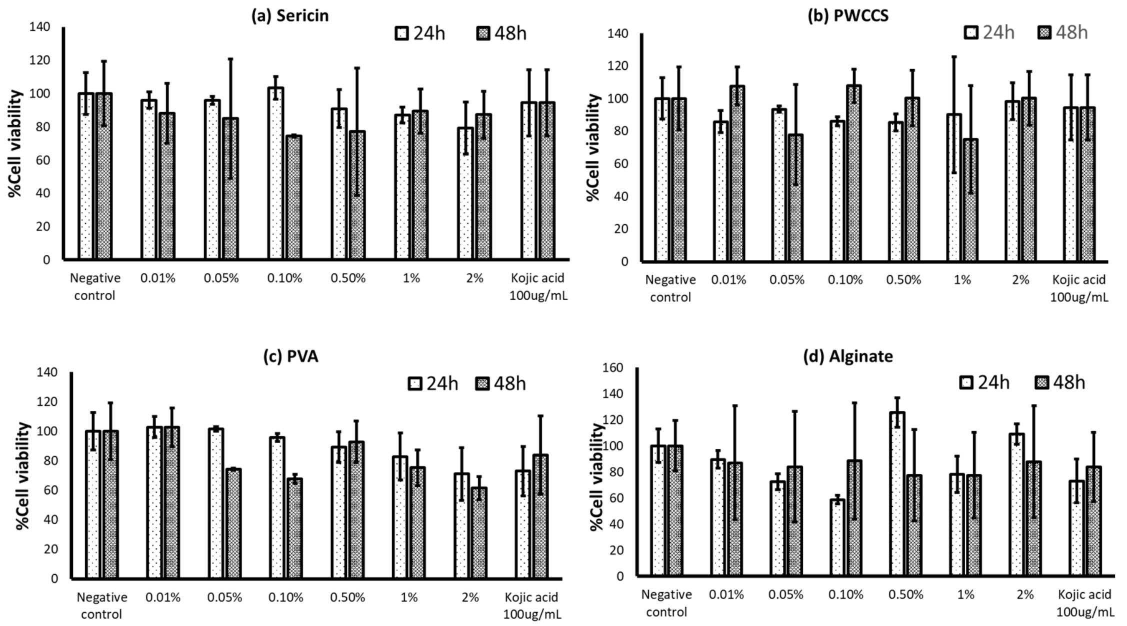

Figure S2: Effects of sericin, PWCCS, PVA and alginate on cell viability of B16F10 cells after treatment for 24, 48 h.

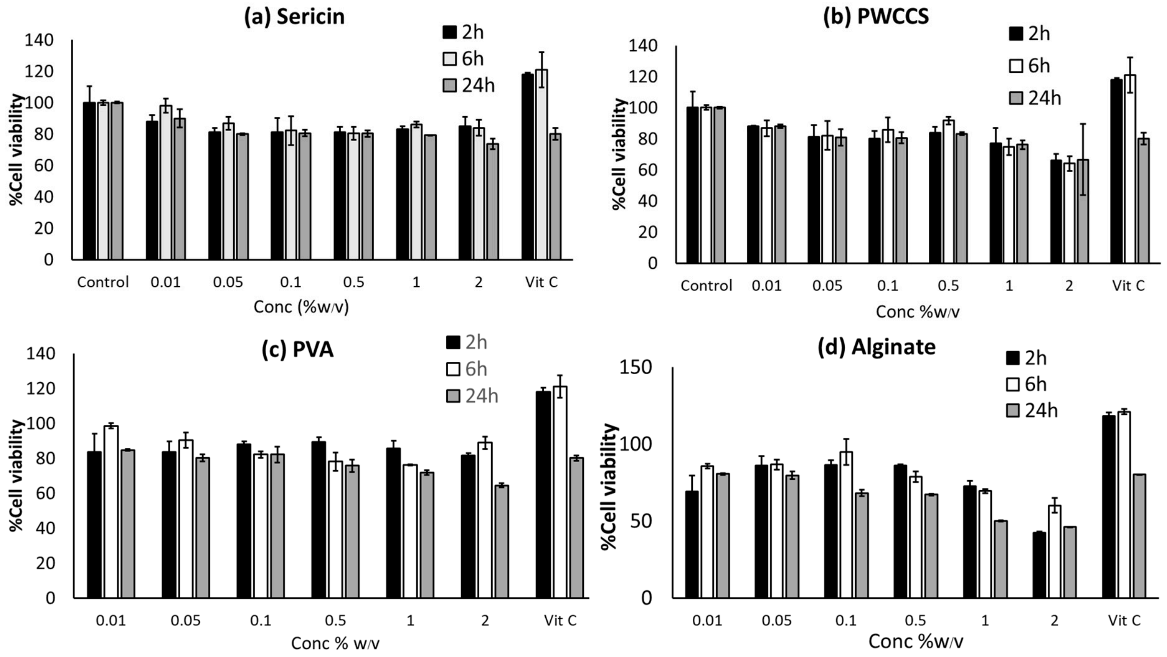

Figure S3: Effects of sericin, PWCCS, PVA and alginate compared with ascorbic acid C 25 μg /mL (Vit C) for cell viability on HaCaT cells after treatment for 2, 6 and 24 h. Data represent the mean ± SEM of three replicates.

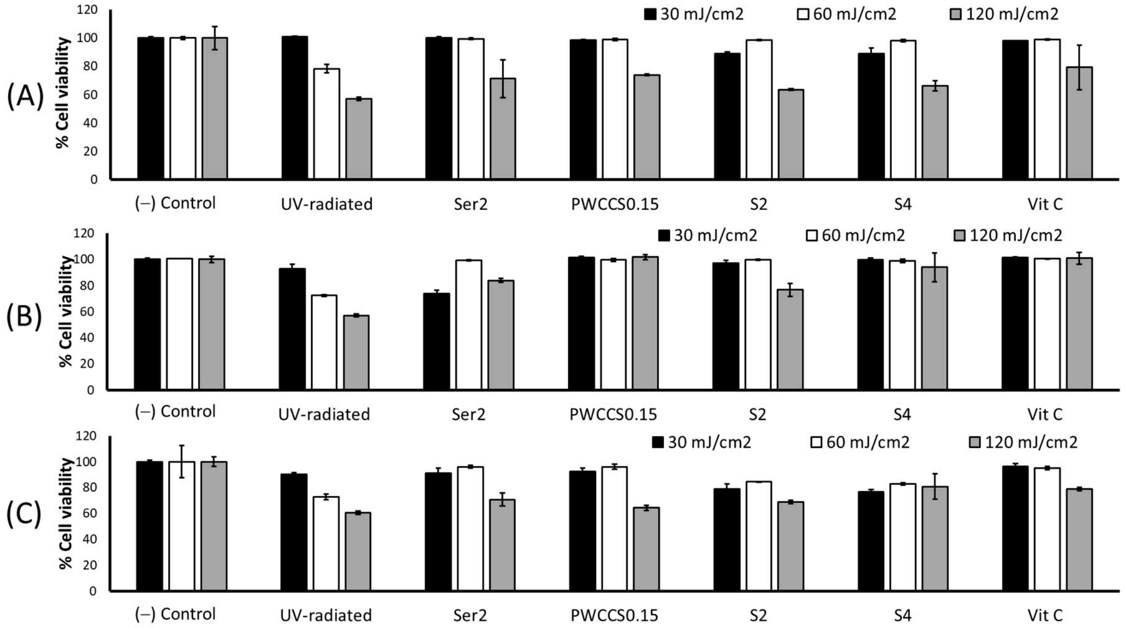

Figure S4: Effects of UV-irradiation for various time of formulation pretreatment of 2%sericin (Ser2), 0.15%PWCCS (PWCCS0.15), formulations (S2 and S4) and ascorbic acid 25 µg/mL (Vit C) on HaCaT cells (A) 2 h pretreatment formulations (B) 6 h pretreatment formulations and (C) 24 h pretreatment formulations. Data represents the mean ± SEM of three replicates.

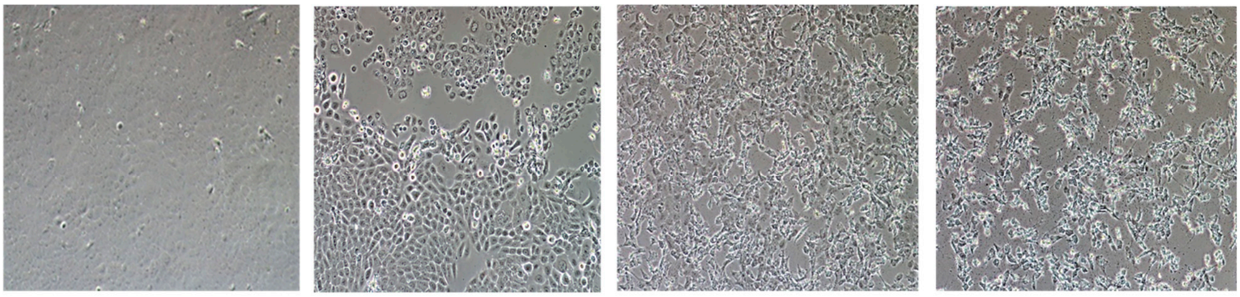

(A) Untreated cells (B) UVB 30 mJ/cm^2) (C) UVB 60 mJ/cm^2 (D) UVB 120 mJ/cm^2

Figure S5: Morphology of UVB irradiation on HaCaT cells: (A) Untreated cells, (B) Cells irradiated with UVB 30 mJ/cm^2, (C) Cells irradiated with UVB 60 mJ/cm^2, (D) Cells irradiated with UVB 120 mJ/cm^2.

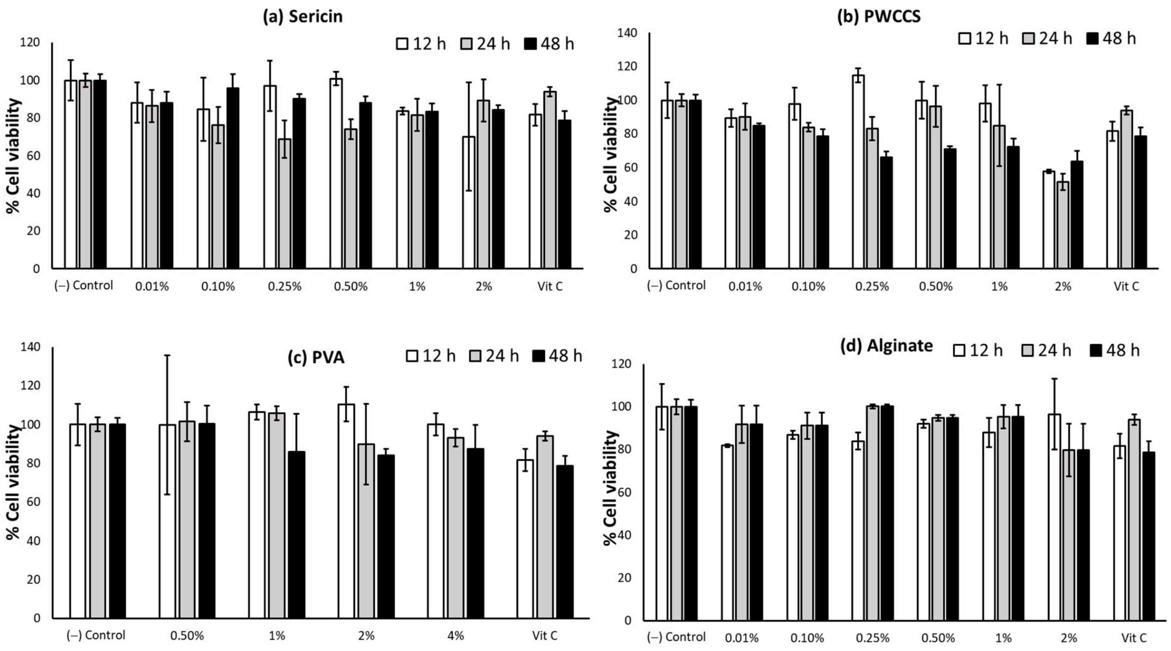

Figure S6: Effects of sericin, PWCCS, Alginate, and PVA on cell viability of NHDF cells after 12, 24, and 48 h.

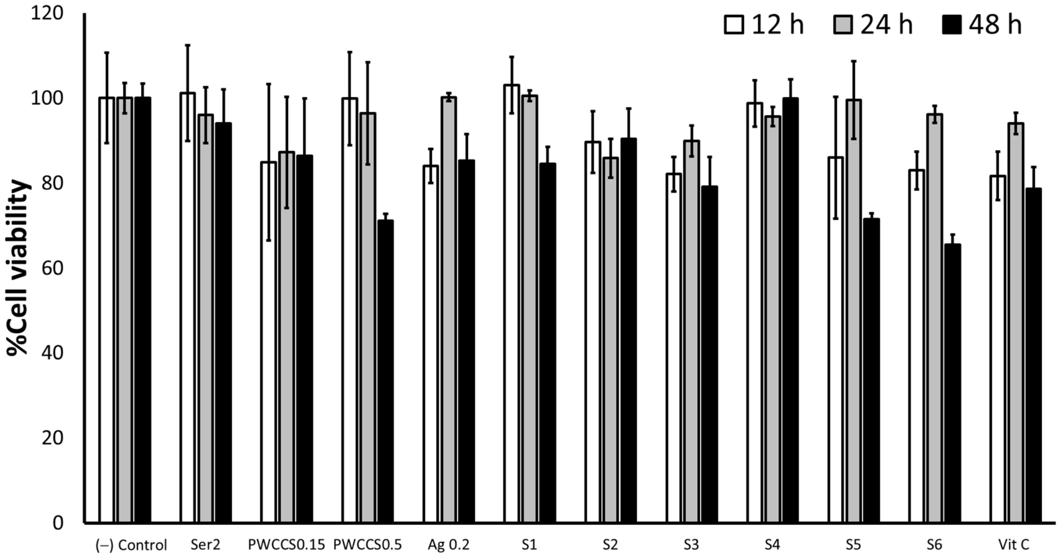

Figure S7: Viability of NHDF cells exposed to 2%sericin (Ser2), 0.15%PWCCS (PWCCS0.15), 0.2%alginate (Ag0.2), 25 µg /mL ascorbic acid (Vit C), formulations S1-S6 for 12, 24 and 48 h. Data represent the mean ± SEM of three replicates.
